# The Treatment of Tobacco Amblyopia by Acetyl-Choline

**Published:** 1936

**Authors:** B. H. Cragg

**Affiliations:** House Surgeon, Bristol Eye Hospital


### THE TREATMENT OF TOBACCO AMBLYOPIA BY ACETYL-CHOLINE.

BY

B. H. CRAGG, L.M.S., M.D.,

*House Surgeon, Bristol Eye Hospital.*

---

Since 22nd July, 1936, five patients, all males, suffering from tobacco amblyopia have been treated at the Bristol Eye Hospital by the intramuscular injection of "pragmoline," acetyl-choline bromide. This drug is available in two doses, dose A being 0·03 grammes, and dose B 0·125 grammes.

Each patient first received two injections of dose A with one clear day's interval between, and then a daily injection of dose B until no further treatment was considered necessary, or until improvement ceased. In addition, the patients 4 and 5 were given physostigmine sulphate gr. $\frac{1}{150}$ daily by mouth, following the suggestion that the action of acetyl-choline is enhanced by minute doses of this drug.

It is hoped by this method to accelerate the treatment of tobacco amblyopia.

The results in these cases are encouraging: treatment by this method gives more rapid improvement in vision, the patient in most cases realizing that over-indulgence in tobacco has been the cause of his defective vision.

**Case 1.**—Age 72. This man complained of failing vision over a period of five months. He reported on 9th June, 1936, at which time his vision was

R. $\frac{2}{60}$. L $\frac{2}{60}$.

Six weeks later there was no improvement, although he had stopped smoking.

He was admitted on 22nd July, and received ten injections. On discharge, two weeks later, his vision was

R. $\frac{6}{18}$ partly. L. $\frac{6}{12}$.

Two months later vision showed no further change.

**Case 2.**—Age 65. This patient reported on 31st July, complaining of defective vision. Vision

R. $\frac{1}{60}$. L. $\frac{1}{60}$.

He was admitted and received nine injections. On discharge, three weeks later, vision was

R. $\frac{6}{6}$ partly. L. $\frac{6}{6}$ partly.

He has failed to report since then.

**Case 3.**—Age 66. This patient attended in 1933, at which time his vision was

R. $\frac{6}{27}$ ; J $\frac{10}{12}$. L. $\frac{6}{36}$ ; J $\frac{12}{12}$.

He was advised to stop smoking, but did not do so. On admission on 25th August, 1936, his vision was slightly worse. He was treated for four weeks, receiving seventeen injections. At the time of discharge his vision was

R. $\frac{6}{18}$ ; J $\frac{4}{12}$ (slowly). L. $\frac{6}{24}$ ; J $\frac{4}{12}$ (slowly).

His fundi, in addition to signs of arteriosclerosis, revealed the presence of fine hæmorrhages.

Two months later his vision was

R. $\frac{6}{18}$ partly ; J $\frac{6}{12}$ slowly. L. $\frac{6}{24}$ partly ; J $\frac{4}{12}$ slowly.

This patient has resumed smoking.

**Case 4.**—Age 60. This patient had noticed failing vision for six weeks. On 24th September, 1936, vision was

R. $\frac{6}{24}$ partly ; J $\frac{12}{12}$. L. $\frac{6}{18}$ partly ; J $\frac{12}{12}$.

He was admitted on 25th September, and discharged on 21st October, and received during that time seventeen injections. On discharge his vision was

R. $\frac{6}{18}$ partly ; J $\frac{4}{12}$. L. $\frac{6}{9}$ partly ; J $\frac{2}{12}$.

He has not reported since discharge.

**Case 5.**—Age 62. This patient had noticed failing vision for six weeks. On 28th September, 1936, vision was

R. $\frac{6}{12}$ ; J $\frac{10}{12}$. L. $\frac{6}{12}$ ; J $\frac{8}{12}$.

He was admitted on 30th September, and discharged on 21st October, having received thirteen injections. His vision on discharge was

R. $\frac{6}{6}$ partly ; J $\frac{1}{12}$. L. $\frac{6}{6}$ partly ; J $\frac{1}{12}$.

He reported a month later, and there was then no change in vision.

I am indebted to the honorary surgeons of the hospital for permission to publish these cases.

